# Society Meetings

**Published:** 1901-04

**Authors:** 


### SOCIETY MEETINGS.

The Central New York Homeopathic Medical Society held its fiftieth annual meeting at Syracuse, Thursday, March 21, 1901. The officers are: Volney A. Hoard, of Rochester, president; Josephine Howland, of Auburn, vice-president; S. L. Guild-Leggett, of Syracuse, secretary and treasurer. Dr. E. P. Hussey, of this city, was the toastmaster at the banquet held in the evening.

The Buffalo Academy of Medicine held meetings during the month of March, 1901, as follows:

*Quarterly Meeting.*—Tuesday evening, March 19th. The Section on Pathology provided the following program: A report of recent investigations of cancer, H. R. Gaylord. A case of organic epilepsy; clinical and pathological report, Henry P. Frost.

*Section on Ophthalmology, Otology, Rhinology and Laryngology.*—Monday evening, March 18th. Program: The making and fitting of spectacles and eye-glasses from the optician's standpoint, Mr. P. H. Meyer. Fractures of the nose, Marshall Clinton. Report of a case of emergency tracheotomy, George F. Cott.

*Section on Medicine.*—Tuesday evening, March 12th. Program: The racial factor in hysteria, Julius Ullman. Hysterial aphonia, George F. Cott. Discussion by William C. Krauss. The $x$-ray in general practice, with stereopticon views, A. W. Bayliss.

*Section on Surgery.*—Tuesday evening, March 5th. Program: Surgical mixed pickles, Marcel Hartwig. Transplantation of tendons, J. Hilton Waterman.

---

An International Congress of Nurses will be held in Buffalo, September, 1901, during the time of the Pan-American exposition, to cement and strengthen the national and international organisations which the nurses of this country, Great Britain, the English Colonies, Denmark and Holland have for the last few years been intent upon developing. The nurses of the English speaking countries also have in view the attainment of state examination and registration.

The national association of the United States has now become affiliated with the National Council of Women, as the others purpose doing in their respective countries.

The president of the International Council of Nurses is Mrs. Bedford Fenwick, of London, and the vice-presidents are the presidents of the respective national councils. The executive officers of the Buffalo congress are the officers of the American Society of superintendents of training schools for nurses, those of the National Alumnæ of schools for nurses, of the Buffalo Nurses' Association, and the American councillors of the international council of nurses.

The chairman of the congress is Miss McIsaac, superintendent of nurses in the Illinois Training School, Chicago, and the secretary is Miss Banfield, superintendent of the Polyclinic Hospital, Philadelphia.

---

The Association of Medical Officers of the Army and Navy of the Confederacy will convene in Memphis, Tenn., May 28-30, 1901, during the meeting of the Confederate Reunion. All surgeons, assistant surgeons, acting assistant surgeons, or contract physicians and hospital stewards, in the Army and Navy of the Confederate States, and all regular physicians who served honorably in any capacity in the Confederate States Army and Navy, and all regular physicians who are sons of confederate veterans, are eligible to membership.

